# A narrative review on cost considerations in early intervention for deaf and/or hard-of-hearing children in Africa

**DOI:** 10.1093/heapol/czaf074

**Published:** 2025-10-08

**Authors:** Katijah Khoza-Shangase

**Affiliations:** Department of Audiology, School of Human and Community Development, University of the Witwatersrand, Johannesburg, South Africa, Private Bag 3, WITS, 2050

**Keywords:** early intervention, deaf and/or hard-of-hearing, health financing, access to care, Africa, disability policy

## Abstract

Early intervention (EI) is essential for the language, social, and educational development of deaf and/or hard-of-hearing (DHH) children. In African countries, however, the implementation of EI remains significantly constrained by cost considerations and systemic service gaps. This narrative review synthesizes findings from 26 peer-reviewed publications to explore how cost influences access to and sustainability of EI services in Africa. Seven interrelated themes were identified: (i) high out-of-pocket expenses that limit family access to services; (ii) inadequate public funding and heavy reliance on private or donor sources; (iii) cost-effectiveness of early screening and intervention when delivered at scale; (iv) lack of integrated cost data in national health planning; (v) inequitable access to hearing technologies due to procurement and pricing challenges; (vi) opportunities for system-level enablers such as intersectoral collaboration, task-shifting, and community-based delivery; and (vii) structural cost drivers unique to African contexts, including fragmented systems and infrastructure disparities. The findings highlight the need to embed economic evidence into policy planning, establish pooled procurement and subsidy schemes to reduce device costs, and integrate EI services into national insurance and essential health benefit packages. Culturally responsive, community-delivered models, supported by sustainable public financing and regional collaboration, are critical to ensure equity and long-term impact. Addressing these cost-related barriers through coordinated policy and system reforms will be key to achieving universal, inclusive, and sustainable EI services for DHH children in Africa.

Key messages

- Cost-related barriers—including high out-of-pocket expenses and fragmented financing—are a major obstacle to EI for DHH children in Africa.
- Systemic drivers such as donor dependency, limited insurance coverage, and poor access to assistive devices perpetuate inequities and require urgent policy reform.
- Embedding cost analyses into health planning and integrating EI into national insurance schemes can improve financial protection and sustainability.
- Investing in culturally responsive, community-based EI models offers an opportunity to strengthen equity and advance universal health coverage goals.

## Introduction

Globally, a substantial number of children require early intervention (EI) to address disabling hearing impairment (World Health Organisation-WHO 2025). Hearing loss is not only a clinical issue, but its impact extends beyond auditory perception, affecting speech, language acquisition, cognitive development, educational attainment, and overall socio-emotional well-being (Khoza-Shangase 2021, Haukedal et al. 2022, Casoojee et al. 2024, Porcar-Gozalbo et al. 2024). Notably, the prevalence of disabling hearing loss in children is disproportionately higher in low- and middle-income countries (LMICs), including those in Africa (WHO 2025). According to the World Health Organization, over 60% of hearing loss in children in LMICs is preventable, yet most remain undiagnosed and untreated. For instance, in Kenya, the occurrence of hearing loss in children is considerably greater compared to high-income nations (Ndegwa et al. 2024). This elevated prevalence in LMICs highlights the critical need for effective and affordable early intervention programmes to mitigate the potential long-term adverse effects on affected children and their communities. The consequences of inaction are both individual and societal, as unaddressed hearing loss contributes to educational exclusion, poor job prospects, and increased health system burdens (WHO 2024). This public health issue transcends individual burden, requiring policy-level responses that integrate hearing care into broader health system strengthening in LMICs.

Early intervention plays a pivotal role in shaping the communication abilities and overall developmental trajectory of children who are deaf and/or hard-of-hearing (DHH) (Casoojee et al. 2025). Research indicates that identification and intervention within the first six months of life are crucial for these children to achieve language development comparable to their hearing peers (Grey et al. 2022). Furthermore, enrolment in EI programmes has been shown to foster improved vocabulary and reasoning skills by the age of five. The evidence strongly suggests that timely intervention can significantly narrow the developmental gap between DHH children and their hearing counterparts, emphasizing the urgency of early detection and ensuring access to necessary services. Missing this critical window results in cascading disadvantages that are often more complex and costly to remediate later in life.

However, translating global evidence into practice within African contexts presents distinct and deeply entrenched challenges (London et al. 2020). The African context presents a unique set of sociocultural and systemic challenges that necessitate the development of tailored approaches to EI (Khoza-Shangase 2025). Several barriers impede the implementation of effective EI programmes, including widespread poverty, limited government investment in healthcare infrastructure, and a high overall burden of disease, which often relegates hearing impairment to a lower priority (Khan and Joseph 2020, Khoza-Shangase and Maluleke 2025). Additionally, a significant challenge lies in the lack of specialized knowledge and the limited capacity to provide the necessary services for DHH children (Pillay et al. 2020, Wonkam-Tingang et al. 2021, Baratedi et al. 2022, Petrocchi-Bartal et al. 2025). Prevailing social beliefs, stigma associated with disability, and general misunderstandings about ear and hearing disorders further complicate the achievement of EI goals (Wonkam-Tingang et al. 2021, Khoza-Shangase 2025). These multifaceted contextual challenges highlight the importance for African-specific EI strategies that are both cost-conscious and culturally aligned.

EI strategies must be grounded in sociocultural relevance and adaptable to the resource-constrained realities of many African countries (Khoza-Shangase 2025). Among the most pressing and cross-cutting challenges encountered in the African context is the critical role of cost considerations in the provision of EI services. Economic constraints pose a substantial barrier to accessing essential diagnostic tools, hearing aids, and consistent follow-up care (Blazer et al. 2016, Waterworth et al. 2022). Budgetary limitations often affect health policy decisions, directly impacting the availability and accessibility of EI services. Furthermore, the lack of dedicated financing for ear and hearing care (EHC) contributes to high costs for interventions, thereby restricting access to necessary hearing aids for a large proportion of those who could benefit. Even when policies exist, their implementation is frequently undermined by cost-related barriers, rendering services ineffective or inaccessible to most of the population (Petrocchi-Bartal et al. 2025). The pervasive influence of cost on the implementation and utilization of EI services in Africa necessitates a thorough examination of the economic landscape to identify both barriers and opportunities for innovation.

In African contexts, the intersection of economic constraints and cultural perceptions of disability necessitates not only cost-sensitive but also culturally grounded interventions. Traditional support structures, community-based care models, and Afrocentric frameworks of wellness and disability must be considered when designing EI services (Khoza-Shangase 2025). Moreover, Africa's colonial legacy continues to influence service delivery models, which are often imported and ill-suited to the continent’s realities (Somerville 2017). Addressing cost without contextualizing cultural and systemic realities limits the effectiveness and acceptability of interventions. This review therefore embraces a decolonial lens to cost considerations, framing them within Africa’s historical, structural, and sociopolitical realities.

This narrative review aims to explore the multifaceted cost considerations associated with EI for DHH children within the African context. The objectives of this review are to highlight the key barriers and enablers related to the affordability and accessibility of EI services, and to ultimately offer recommendations for the development of sustainable and culturally aligned interventions that can effectively address the needs of this vulnerable population. To contextualize these challenges, this review systematically examines how cost considerations affect the availability, accessibility, and sustainability of EI services across the African continent. By drawing from the growing body of African-centred evidence, this review contributes to a nuanced understanding of the economic, systemic, and cultural levers necessary to advance equitable early hearing care. It examines cost considerations through a health systems lens, synthesizing evidence to inform health policy decisions around the integration, financing, and delivery of EI services for DHH children in Africa. It aims to support decision-makers by identifying cost-related bottlenecks, enabling factors, and strategic entry points for investment.

## Methods

This review adopts a narrative review design with systematic elements to synthesize existing evidence from peer-reviewed literature concerning cost considerations in EI for DHH children in Africa. A narrative review approach was deemed appropriate due to its flexibility in addressing complex, context-specific issues that span multiple disciplines (Ferrari 2015), including audiology, public health, education, and economics. Also, a narrative approach was chosen because the literature on cost considerations in EI for DHH children in Africa is heterogeneous in design, scope, and methodological quality, making meta-analysis or strict systematic synthesis inappropriate. At the same time, to enhance rigour and transparency, structured elements commonly used in systematic reviews were incorporated, including comprehensive multi-database searching, predefined inclusion and exclusion criteria, a PRISMA-style flow diagram to document study selection, and structured evidence extraction into tabulated form. This hybrid approach allowed for the identification of broad patterns and thematic synthesis across diverse study types, while maintaining the flexibility needed to interpret findings considering African contextual and policy realities. This methodology allowed for a thematic synthesis of diverse study types, enabling the identification of patterns, contradictions, and gaps that may not emerge from systematic or meta-analytic approaches.

The search strategy involved a comprehensive and iterative search of the following electronic databases: PubMed, Scopus, Web of Science, and Google Scholar. The search terms employed encompassed various combinations of keywords related to early intervention (e.g. ‘early intervention,’ ‘early hearing detection and intervention,’ ‘EHDI’), hearing loss (e.g. ‘hearing loss,’ ‘deaf,’ ‘hard-of-hearing,’ ‘hearing impairment’), the target population (e.g. ‘children,’ ‘infants’), cost-related aspects (e.g. ‘cost,’ ‘economics,’ ‘affordability,’ ‘funding,’ ‘resource allocation’), and the geographic focus (‘Africa’ and individual country names). The search was limited to peer-reviewed literature published between 2000 and 2024 to ensure the inclusion of contemporary, policy-relevant research. The year 2000 was chosen as the starting point to capture contemporary evidence reflecting more recent policy developments, advances in early hearing and intervention (EHDI) technologies, and shifts in health financing priorities relevant to the African context. To enhance transparency, the full search strategy with example search terms for PubMed is provided in Supplementary Appendix S1 (Search Strategy (Example: PubMed)—Supplementary File), with equivalent terms adapted for Scopus, Web of Science, and Google Scholar.

The inclusion criteria for this review were as follows: publications focusing on EI (defined as interventions initiated before school age) for DHH children; studies conducted in African countries or specifically addressing the African context; studies that included a focus on cost considerations, economic evaluations, funding models, or affordability of EI services; and peer-reviewed literature published in the English language. Publications were excluded if they: (i) primarily focused on hearing screening without discussing cost aspects of subsequent interventions; (ii) were conducted outside Africa; or (iii) comprised grey literature, conference abstracts, or non-peer-reviewed sources.

**Figure 1.**
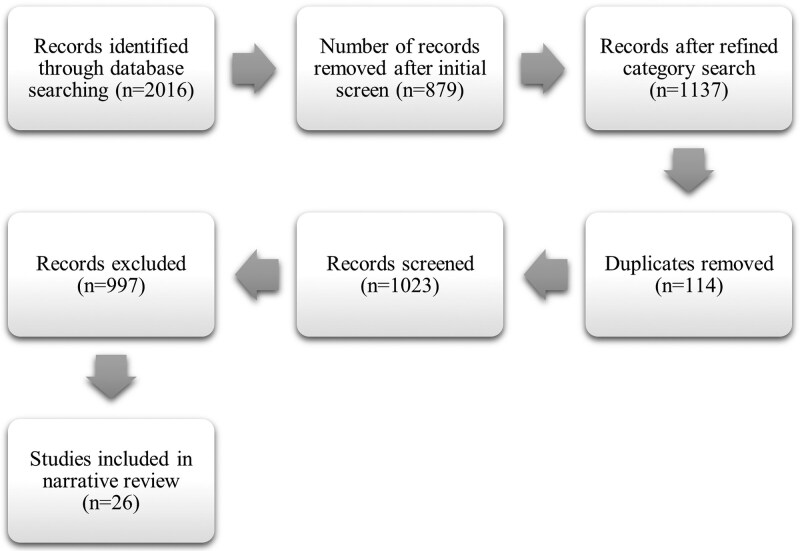
Document selection flow diagram.

A PRISMA-style flow diagram (see Fig. 1) illustrates the article selection process. The initial search across all databases identified 2016 records. To improve relevance, a refined category search was conducted using database-specific filters (e.g. limiting to subject categories such as medicine, public health, audiology, and rehabilitation, and excluding unrelated fields such as engineering or veterinary science). This step reduced the pool to 1137 records. The refinement was necessary to eliminate large volumes of irrelevant records and to focus the review on literature directly addressing EI, cost considerations, and hearing health in human populations. After removing 114 duplicates, 1023 records were screened for eligibility. Of these, 997 were excluded, resulting in 26 studies being included in the final review.

Data extraction was performed using a standardized extraction form designed to ensure consistency in the variables captured across studies. The extracted data were synthesized using a thematic analysis approach. Initially, descriptive summaries of each study were reviewed and coded for cost-related issues, barriers, enablers, and recommendations. Codes were iteratively grouped into broader categories that reflected recurring patterns across the studies. Through constant comparison, these categories were refined into seven overarching themes capturing the multidimensional nature of cost considerations in EI for DHH children. The interpretive approach was both inductive, allowing themes to emerge from the data, and deductive, informed by the review’s guiding questions and existing frameworks on health systems and cost analysis. Reflexivity was maintained throughout to ensure that interpretations were grounded in the evidence while also attentive to the African sociocultural and health policy context. Extracted data included: citation details (author, year, title), country, study design, population characteristics, type of intervention, cost components evaluated (e.g. direct, indirect, intangible), costing methodology (e.g. top-down, bottom-up), main findings related to cost, identified barriers/enablers, funding source, recommendations, and contextual relevance to Africa.

While formal critical appraisal was not undertaken due to the narrative nature of the review, each study was assessed for methodological clarity, relevance to the review objectives (Colquhoun et al. 2014), and consistency with African healthcare and socio-economic contexts (Supplementary Appendix S2—Appraisal of Included Studies—Supplementary File). To strengthen transparency and replicability, a second reviewer (P.M.) independently verified the extracted data for a subset of studies (Hardwicke et al. 2020).

To ensure credibility, transparency, and reliability of the review process, several safeguards were applied. These included systematic documentation of the search strategy and study selection process, use of predefined inclusion and exclusion criteria, and extraction of data with a standardized form. A second reviewer (P.M.) independently verified a subset of extracted data to check for accuracy and consistency. Reflexivity was maintained by considering how researcher positionality and contextual knowledge might influence interpretation. The PRISMA-style flow diagram was used to transparently document the selection process. Together, these safeguards strengthen the trustworthiness of the findings.

As this study was a literature-based narrative review drawing exclusively on previously published, peer-reviewed sources, formal ethical approval was not required. Nonetheless, ethical integrity was maintained throughout the review process. All sources were comprehensively cited to uphold academic integrity and avoid plagiarism. Only publicly available peer-reviewed literature was used to ensure credibility. Objectivity was prioritized in study selection and analysis to minimize bias, and potential limitations in the evidence base were acknowledged. Efforts were also made to incorporate diverse perspectives, identify research gaps, and ensure transparency in reporting and interpretation.

## Results

### Description of included publications

**Figure 2.**
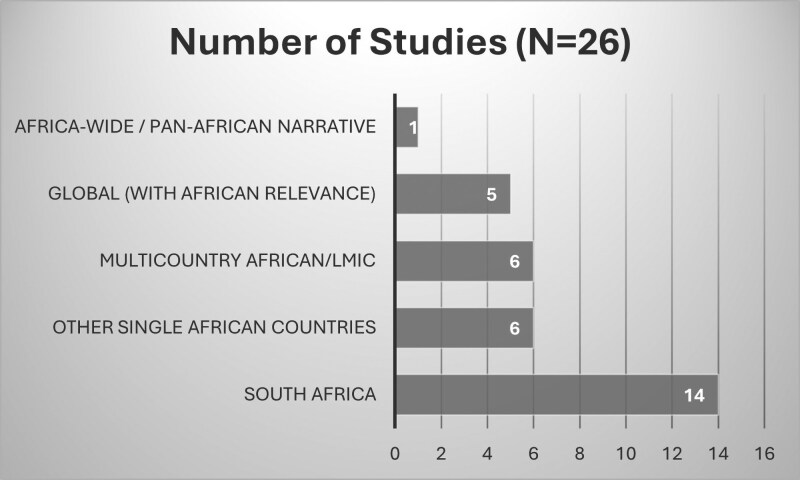
A summary of study characteristics.

A total of 26 peer-reviewed publications were included, spanning diverse study designs such as cost analyses (*n* = 7) (e.g. Olusanya et al. 2009, Kanji and Kara 2013, WHO 2017), narrative/integrative reviews (*n* = 8) (e.g. Swanepoel et al. 2009, Bodington et al. 2021, Khoza-Shangase 2025, Petrocchi-Bartal et al. 2025), qualitative studies (*n* = 5) (e.g. Swanwick et al. 2022, Jesuyajolu et al. 2023, Khoza-Shangase and Bent 2025), economic modelling studies (*n* = 3) (e.g. Baltussen and Smith 2009, Emmett et al. 2015), observational research (*n* = 4) (e.g. Olusanya 2006, Kanji and Kara 2013, Ndegwa 2023, Casoojee et al. 2024), and policy papers (*n* = 3) (e.g. World Health Organization and World Bank 2011, Ndegwa et al. 2024) (see Table 1 for full details of each included study). Studies were geographically distributed across South Africa (*n* = 14; e.g. Swanepoel et al. 2009, Kanji and Kara 2013, Casoojee et al. 2024, Khoza-Shangase and Bent 2025, Petrocchi-Bartal et al. 2025), Kenya (*n* = 2; Ndegwa 2023, Ndegwa et al. 2024), Nigeria (*n* = 3; Olusanya 2006, Olusanya et al. 2009, Jesuyajolu et al. 2023), and Ghana (*n* = 1; Swanwick et al. 2022). The remaining studies comprised global (with African relevance) analyses (*n* = 5) or Africa-wide/Pan-African narrative that explicitly incorporated African data (*n* = 1). This distribution reflects a strong research concentration in Southern Africa and notable gaps in West, Central, and North Africa. A summary of study characteristics is presented in Fig. 2.

**Table 1.** Comprehensive evidence synthesis of cost considerations in early intervention for deaf and/or hard of hearing children in Africa.

C	Citation	Country	Study design	Population	Intervention type	Cost components evaluated	Costing methodology	Main findings	Barriers/enablers	Funding source	Recommendations	Relevance to Africa
1	Störbeck and Young (2016)	South Africa and LMICs	Conceptual chapter	Infants and toddlers with hearing loss	EI in resource-constrained contexts	Not directly evaluated	Conceptual discussion	Reviews practical, cultural, ethical, and systemic EI constraints in LMICs	Limited resources; mismatch with Western EI models	Not applicable	Contextualize EI to align with family and health system realities	Strong relevance—discusses barriers like those in Africa
2	Emmett et al. (2015)	Sub-Saharan Africa	Cost-effectiveness study	Children with profound HL	Cochlear implantation and deaf education	Costs of CI, education, DALYs	Cost-effectiveness using DALYs averted	CI and education shown to be highly cost-effective in SSA	Resource scarcity, infrastructure gaps	Not externally funded	Promote CI and educational investment for children in SSA	High—quantitative justification for cochlear implantation in Africa
3	Fagan and Tarabichi (2018)	LMICs	Commentary	Individuals eligible for CI	Cochlear implantation	Not directly evaluated	Expert opinion	Discusses ethical, policy, and cost barriers to CI access in LMICs	Ethical tension between resource allocation and benefit	Not applicable	Develop national CI strategies considering ethics and access	Medium—informs policy/ethics dialogue for African CI expansion
4	Bodington et al. (2021)	LMICs	Narrative review	Patients eligible for CI	Cochlear implantation	Affordability, logistics, sustainability	Literature synthesis	Highlights feasibility of implementing CI in LMICs despite challenges	Training, maintenance, long-term cost burdens	Not specified	Emphasize local training and phased CI implementation	High—pragmatic guidance for African CI rollout
5	Fagan (2012)	Global focus with emphasis on LMICs, particularly in Africa.	Expert commentary and policy perspective	Patients with ear, nose, and throat (ENT) conditions in developing countries; ENT healthcare providers	Capacity building through training and education in ENT services; development of sustainable ENT healthcare systems.	While specific cost components are not quantified, the article discusses the financial implications of inadequate ENT services, including the costs associated with lack of training, equipment, and infrastructure.	Not applicable	Significant disparity in ENT service provision between developed and developing countries. Developing countries face challenges such as limited training opportunities, lack of equipment, and insufficient infrastructure for ENT services. Collaborative efforts, including training programmes and partnerships with institutions in developed countries, are essential to address these challenges.	‘Barriers’: Limited access to training and education in ENT; scarcity of resources and equipment; inadequate healthcare infrastructure. ‘Enablers’: International collaborations; establishment of training programmes; commitment from local governments and institutions to develop ENT services.	Not specified	Encourage otolaryngologists from developed countries to contribute to training and education in developing countries. Develop sustainable ENT services through capacity building and infrastructure development. Foster global responsibility and partnerships to improve ENT healthcare in resource-limited settings	Highly relevant; the article emphasizes the challenges faced by African countries in providing adequate ENT services and highlights the need for international collaboration to build capacity and improve healthcare outcomes in the region.
6	Kerr et al. (2012)	South Africa	Cross-sectional survey and patient record review	Adults and children who received cochlear implants	Cochlear implantation and associated services	Initial implant system purchase Processor upgrades Batteries and spares Insurance Processor repairs Travel and accommodation Rehabilitation services (especially for children	Costs were categorized into short- and long-term expenses. Consumer Price Index for real-term comparisons over time.	Average 10-year costs: R379,626 for adults; R455,225 for children. Initial implant system purchase was the most substantial cost. Processor upgrades averaged R85,000 every 7 years. Annual costs: batteries and spares (R2,550), insurance (R4,040), processor repairs (R3,000). Travel and accommodation costs were highest in the first two years post-implantation. Children's rehabilitation services averaged R7,200.	‘Barriers’: High initial and ongoing costs, lack of public funding, limited access to implant centres, especially for those living far from urban areas. ‘Enablers’: Potential for improved quality of life and communication abilities; awareness of long-term costs can aid in financial planning.	Not specified	Prospective cochlear implant recipients should be informed about both initial and ongoing costs to ensure long-term sustainability. Financial planning, including savings and insurance, is crucial for managing expenses. Consideration of travel and accommodation costs is important, especially for those residing far from implant centres.	High—provides detailed insights into the financial implications of cochlear implantation in a South African context, highlighting challenges that are applicable to many African countries where public funding is limited and access to specialized services may be constrained
7	Olusanya et al. (2009)	LMICs	Cost-effectiveness analysis	Newborns	Universal Newborn Hearing Screening (UNHS)	Cost per DALY averted	WHO-CHOICE and cost-utility modelling	UNHS is cost-effective across a range of LMICs	Implementation cost, training	Not specified	EHDI should be priority in LMIC health planning	High—supports economic viability of UNHS in Africa
8	Smith (2013)	South Africa	Mixed-methods doctoral dissertation	Individuals with disabling hearing impairment (DHI) in South Africa, encompassing both children and adults.	Assessment of various interventions and services related to DHI, including deaf education, sign language interpretation, hearing aids, cochlear implants, and genetic testing.	Direct costs: Hearing aids, cochlear implants, sign language interpretation services, genetic testing. Indirect costs: Socioeconomic disadvantages, including low income, unemployment, and limited access to education and healthcare services.	The study utilized a combination of survey data, literature reviews, and policy analysis to estimate costs associated with DHI. Specific costing models or economic analyses were not detailed.	Individuals with DHI in South Africa face significant challenges, including inadequate deaf education, high costs of assistive devices, and limited access to services. The cost of sign language interpretation services is prohibitively high for most individuals and institutions. Hearing aids and cochlear implants are often unaffordable, with limited government support and reluctance from medical schemes to cover these costs. Lack of comprehensive disability legislation specifically addressing the needs of individuals with DHI.	‘Barriers’: High costs of assistive devices and services, insufficient government support, lack of comprehensive disability legislation, limited access to quality education and healthcare services. ‘Enablers’: Presence of world-class universities that can contribute to research and development, potential for international collaborations to improve services and reduce costs.	Not specified	Develop and implement comprehensive disability legislation Increase government support and funding for assistive devices and services Promote research and development to improve the affordability and accessibility of assistive technologies	High—the study provides an in-depth analysis of the challenges faced by individuals with DHI in South Africa, offering insights that can inform policies and practices across the African continent to improve access to and affordability of EI services
9	World Health Organization and World Bank. (2011)	Global (includes LMICs and African contexts)	Global policy report with synthesized evidence	Persons with disabilities, including children	EI and rehabilitation services	Direct costs (medical care, assistive devices); Indirect costs (productivity loss, exclusion from education/employment)	Literature synthesis; cross-country economic data from disability-related research	EI can reduce long-term economic burden EI yields social and economic benefits.	Barriers: High cost of services, lack of insurance, insufficient public funding, stigma, inaccessible infrastructure. Enablers: Community-based rehabilitation, inclusive education, and public financing.	Not explicitly stated	Governments should invest in affordable, accessible EI and rehabilitation; integrate disability into mainstream health and education systems.	Highly relevant—emphasizes the need for EI to prevent long-term economic exclusion of children with disabilities in LMICs, including African countries.
10	Baltussen and Smith (2009)	Africa and Asia	Cost-effectiveness modelling	All ages	Screening, Hearing Aids, Otitis Media Treatment	Cost per DALY averted	WHO-CHOICE simulation modelling	Otitis media treatment highly cost-effective (<I$63/DALY); hearing aids and screening cost-effective (I$1000–1600/DALY)	Passive screening and access to devices identified as barriers/enablers	Not stated	Prioritize otitis media treatment; scale up affordable screening and hearing aid access	Continental evidence supports scalable interventions in African contexts
11	Maluleke (2022)	South Africa	Conceptual analysis and policy advocacy	Infants and young children with hearing impairment; policymakers; audiologists	EHDI Programmes	Need for comprehensive economic evaluations of EHDI programmes, focusing on cost-effectiveness analyses (CEA) to assess the value of EI services. Specific cost components are not detailed but are implied to include screening, diagnosis, intervention, and long-term outcomes	Importance of applying cost-effectiveness analysis frameworks to EHDI programmes to generate robust evidence for policy advocacy and resource allocation. It does not present original empirical data but advocates for the use of CEA in evaluating EHDI services	There is a lack of comprehensive economic evaluations of EHDI programmes in South Africa. Implementing cost-effectiveness analyses can provide evidence to support the inclusion of EHDI services in health policy and funding decisions. Economic evaluations can demonstrate the long-term benefits and cost savings of EI for hearing impairment	‘Barriers’: Limited awareness among policymakers about the economic benefits of EHDI; lack of economic evaluation studies; resource constraints in the healthcare system. ‘Enablers’: Growing recognition of the importance of EI; existing frameworks for cost-effectiveness analysis; alignment with Sustainable Development Goals and universal health coverage initiatives.	Not stated	Conduct comprehensive cost-effectiveness analyses of EHDI programmes to inform policy and funding decisions. Advocate for the inclusion of EHDI services in national health agendas and resource allocation frameworks. Enhance the capacity of audiologists and researchers to perform economic evaluations of hearing health services.	High—addresses the need for economic evidence to support EHDI programmes in resource-constrained settings, emphasizing the importance of integrating such services into broader health and development agendas across the African continent
12	Ndegwa et al. (2024)	Kenya	Policy brief	Children	EHDI Programmes	Cost of services, accessibility of assistive technology	Cost-benefit insights (1.67 ID return per 1 ID invested)	UNHS and assistive tech offer strong returns on investment	Equipment costs, HR shortages; supportive policy pathways	Not stated	Increase investment in assistive tech and strengthen policy implementation	Country-specific cost-effectiveness evidence from Kenya
13	Khoza-Shangase (2025)	Africa	Narrative review	DHH Children and Families	Family-Centred EHDI	Economic constraints to implementation	Thematic synthesis	Systemic and economic challenges hinder EHDI implementation	Fragmented systems, cultural diversity; enablers include community support and digital tech	Not stated	Strengthen collaboration, tech solutions, and culturally appropriate policy	Advocates for Afrocentric, decolonized EHDI planning
14	WHO (2017)	Global (including LMICs)	Cost analysis	Global Population	Prevention, Screening, Hearing devices	Direct health costs, education loss, productivity loss	Global burden model	Global hearing loss costs: US$750–790 billion; prevention and EI are cost-effective	High LMIC burden and unmet device need	WHO	Invest in early identification, hearing technology, and prevention strategies	Global evidence reinforces African investment arguments
15	Swanepoel et al. (2009)	South Africa	Narrative review	Infants with hearing loss	EHDI Services	Resource constraints (not costed)	Descriptive summary of system challenges	Majority of South African infants lack access to EHDI services	Workforce and infrastructure shortages	Not stated	Integrate EHDI into national health systems	Highlights national implementation challenges in SA
16	Casoojee et al. (2024)	South Africa	Observational study	Children with hearing loss	LSL-SA vs. TSLT in EI	Relative economic impact on outcomes (not costed formally)	Programme outcome comparison	LSL-SA showed better child outcomes but was more costly and private-sector driven	High costs and inequity in service access	Not stated	Promote public-private partnerships and strengthen public EI capacity	Demonstrates real-world outcome and cost trade-offs
17	Swanwick et al. (2022)	Ghana	Qualitative	Caregivers of DHH Children	Early Childhood Care and Education (ECCE)	Resource availability, caregiver burden	Thematic analysis of caregiver interviews	ECCE access is limited due to barriers like lack of materials and specialized support	Need for multimodal strategies and enabling environments	British Academy, GCRF	Support for ECCE access, inclusive curricula, caregiver training	Country-specific early care and education challenges
18	Kanji and Kara (2013)	South Africa	Prospective observational	Newborns in a public hospital	Newborn hearing screening (NHSP)	Staff time, equipment, programme time	Time–cost tracking and descriptive costing	NHSP was feasible with a low average cost of R35.56 per infant screened.	Barriers: human resource constraints. Enablers: integration into existing maternity services.	Not specified	Integrate NHSP into public-sector maternity services to ensure sustainable access.	Provides local cost data supporting NHSP feasibility in African contexts.
19	Baltussen and Smith (2009)	Africa and Asia	Cost-effectiveness modelling	All ages	Screening, hearing aids, otitis media treatment	Cost per DALY averted	WHO-CHOICE simulation modelling	Otitis media treatment was highly cost-effective (<I$63 per DALY averted); hearing aids and screening were cost-effective (I$1000–1600 per DALY).	Barriers: limited access to devices, passive screening.	Not stated	Prioritize otitis media treatment and expand affordable hearing aid and screening access.	Provides robust continental-level evidence for scalable, cost-effective hearing interventions.
20	Sharma et al. (2019)	Global	Systematic review	Children	Childhood hearing screening	Programme costs and outcomes	Review of economic evaluations (varied methods)	Screening generally cost-effective, but methodological weaknesses limited evidence strength.	Barriers: inconsistent methodology; lack of comparability.	Not specified	Strengthen rigour, transparency, and reporting in economic evaluations.	Offers methodological guidance to strengthen African hearing health economic research.
21	Emmett and Francis (2015)	Global (includes LMICs)	Policy and cost review	DHH children and adults	Screening and intervention services	Healthcare, education, productivity losses	Literature synthesis and economic modelling	Hearing loss interventions were highly cost-beneficial in LMICs.	Barriers: underfunding and data gaps.	Not specified	Embed hearing health into national health financing and policy frameworks.	Provides strong economic rationale for integrating hearing care into African health policy.
22	Petrocchi-Bartal et al. (2025)	South Africa	Integrative review	Children with hearing loss	Early Hearing Detection and Intervention (EHDI)	Not specified	Narrative synthesis	Identified gaps between policy and practice in EI service delivery.	Barriers: lack of standardized policy implementation and training.	Not specified	Align EI services with local contexts through integrated models and training.	Highlights disconnect between policy intent and service reality, relevant across African contexts.
23	Olusanya (2006)	Developing countries (includes Africa)	Narrative review	Infants and children	Early Hearing Detection and Intervention (EHDI)	Not directly evaluated	Literature synthesis	Documented lack of newborn hearing screening in LMICs and proposed feasibility strategies.	Barriers: low political will, poor health system integration.	Not specified	Incorporate EHDI into maternal and child health services.	Provides foundational arguments for scaling EHDI in Africa.
24	Ndegwa (2023)	Kenya	Cross-sectional qualitative study	30 children with cochlear implants (data from caregivers)	Cochlear implantation	None	Caregiver interviews and file reviews	Median age at suspicion ∼2–3 years; average 2-year delay to implantation. Barriers: no newborn hearing screening, high CI cost, low awareness.	Barriers: systemic and financial. Enablers: NGO support.	Institutional review (no specific funding)	Advocate for newborn hearing screening, reduce CI costs, and increase community education.	Demonstrates delays and systemic barriers to CI in East Africa, applicable across the continent.
25	Jesuyajolu et al. (2023)	Nigeria	Scoping review	3 studies; 25 CI recipients in Nigeria	Cochlear implantation	None	Literature review (PRISMA-ScR)	CI recipients mostly affected by febrile illness and meningitis. Barriers included high CI cost, lack of rehabilitation, few trained staff, and skepticism about CI.	Barriers: economic and systemic constraints. Enablers: small successful CI programmes.	None reported	Subsidize CI, expand multidisciplinary CI teams, and increase public awareness.	Highlights Nigeria’s CI challenges, reflecting barriers widely seen across LMICs.
26	Khoza-Shangase and Bent (2025)	South Africa	Mixed-method (Q-method survey + interviews)	9 parents of children with cochlear implants	Cochlear implantation decision-making	None (qualitative/attitudinal)	Q-methodology and thematic analysis	Two parental groups: (i) ‘CI essential’ prioritizing speech outcomes; (ii) ‘CI conditional’ with context-based concerns. Themes: financial barriers, clinician influence, stigma.	Barriers: socioeconomic inequity, limited infrastructure, stigma. Enablers: parental recognition of benefits, professional support.	University funding	Expand publicly funded CI programmes, provide culturally sensitive counselling, and raise awareness to reduce stigma.	Provides insights into sociocultural and financial dynamics influencing CI uptake, relevant across African contexts.

CI, cochlear implants; EI, early intervention; DALY, disability-adjusted life year; SSA, sub-Saharan Africa; LMICs, low- and middle-income countries; UNHS, universal newborn hearing screening; EHDI, early hearing detection and intervention; ROI, return on investment; ECCE, early childhood care and education; NHSP, newborn hearing screening programme; GCRF, global challenges research fund; DHH, deaf and/or hard-of-hearing; LSL-SA, listening and spoken language—South Africa; TSLT, total communication/sign language therapy (context-specific, often referred to as ‘Total Communication Sign Language Therapy’); ENT, ear, nose, and throat.

Interventions studied include universal newborn hearing screening (UNHS), cochlear implants, hearing aids, family-centred EI (FCEI) models, and early childhood education. Recent contributions also highlight parental decision-making processes around cochlear implantation and their financial/cultural dimensions (Khoza-Shangase and Bent 2025), as well as policy–practice gaps in early intervention delivery (Petrocchi-Bartal et al. 2025). Understanding the distribution of study designs, geographic focus, and the specific interventions examined provides essential context for interpreting the findings of this review and identifying areas where further research may be warranted.

### Evidence table

The evidence table summarizing key data extracted from the included publications is presented in Table 1. The evidence table serves as a foundation for the thematic synthesis that follows, illuminating the multidimensional nature of cost-related issues.

### Thematic analysis findings

The thematic analysis of the extracted data revealed seven recurring themes related to cost considerations in EI for DHH children in Africa.

#### Theme 1: out-of-pocket costs and financial protection gaps in EI services

A central theme was the significant cost considerations to accessing EI services. The high costs associated with screening, diagnosis, the purchase and maintenance of hearing aids and cochlear implants, ongoing therapy sessions, and specialized educational support place a substantial economic burden on families (Fagan 2012, Kerr et al. 2012, Emmett et al. 2015, Casoojee et al. 2024). In South Africa, for example, caregivers report challenges affording hearing aids and transport to clinics (Khoza-Shangase and Lallubhai 2025). Parental decision-making studies also show that financial constraints strongly influence whether families proceed with cochlear implantation, often alongside concerns about stigma and access to long-term rehabilitation (Jesuyajolu et al. 2023, Khoza-Shangase and Bent 2025). The financial strain can be so severe that it dictates whether a child receives the necessary and timely support. This economic burden not only affects access to care but can also impact families’ employment and daily lives, with some caregivers having to make significant sacrifices to manage their child's appointments while minimizing work absenteeism (Syed et al. 2020, Loh et al. 2025). The consistent identification of high costs as a major impediment highlights the urgent need to address the economic challenges faced by families seeking EI for their children. These cost considerations reflect not only individual affordability challenges but also systemic inequities in health and education financing.

#### Theme 2: cost-effectiveness of interventions

Despite these barriers, many studies found EI to be cost-effective. WHO data estimates a positive return on investment for UNHS in LMICs—a return of 1.67 international dollars for every 1 invested in UNHS in these countries (WHO 2017, Ndegwa et al. 2024). Cochlear implantation and deaf education are also shown to be cost-effective when aligned with gross domestic product (GDP) thresholds (Emmett et al. 2015). Providing hearing aids or cochlear implants can lead to significant savings in the long term within these contexts. A cost study in Kenya and other sub-Saharan African countries found deaf education to be cost-effective, and cochlear implants could be if priced appropriately relative to the gross domestic product (Emmett et al. 2015, Ndegwa et al. 2024). In Nigeria, community-based UNHS via immunization clinics yielded low per-case detection costs (Olusanya et al. 2009). South African evidence further demonstrates that newborn hearing screening can be delivered at very low average costs (Kanji and Kara 2013), supporting arguments for its feasibility in LMIC health systems. These findings highlight that while the initial costs of intervention can be high, the long-term economic and social benefits often outweigh these initial investments.

#### Theme 3: fragmented financing models and the need for integrated resource allocation

The review also identified various funding models and resource allocation challenges and strategies. Current funding for EI services in Africa comes from multiple sources, including government allocations, donor support, and significant out-of-pocket expenditures by families (Swanepoel et al. 2009, Fagan and Tarabichi 2018). However, ensuring sustainable funding remains a critical challenge. Many countries lack a dedicated EHC budget (Petrocchi-Bartal et al. 2025). Financial assistance mechanisms, such as government subsidies and NGO-sponsored programmes, are crucial facilitators for lower-income families. Evidence from Kenya highlights that cochlear implantation is largely dependent on NGO or donor support, with delays linked to high costs and lack of systemic subsidies (Ndegwa 2023). The need for sustainable funding models is further emphasized by the fact that a significant percentage of African countries do not have a dedicated budget allocated to ear and hearing care activities, leaving patients to bear the entire cost of treatment and care (WHO 2024). This complex and potentially unstable financial landscape raises the importance of exploring and implementing robust and sustainable funding mechanisms for EI services. Suggested solutions include cross-sectoral budgeting such as financing between ministries of health and education (Kanji 2021) and integrating EHC into the upcoming national health insurance (NHI) (Maluleke 2022).

#### Theme 4: economic impact of hearing loss

The broader economic impact of hearing loss on individuals, families, and national economies is substantial. Globally, unaddressed hearing loss results in significant economic costs annually. These costs include healthcare expenditures, educational support, productivity losses due to unemployment, and societal costs related to social isolation and communication difficulties (Smith 2013, WHO 2017). In Africa, the annual cost of hearing loss is estimated to be billions of dollars (WHO 2024). This significant economic burden provides a strong rationale for investing in EI programmes, not only as a means of improving individual outcomes but also as a long-term cost-saving measure for society. Early intervention can potentially reduce the need for more costly interventions and support services later in life, while also increasing the individual's potential for economic contribution. Quantifying the cost of inaction offers strong economic justification for investment (World Health Organization and World Bank 2011).

#### Theme 5: barriers to implementation beyond cost

Beyond the direct costs of services, the review highlighted several other barriers to implementation beyond cost. In addition to cost considerations, other barriers include equipment shortages, human resource limitations, fragmented referral systems with its disjointed care pathways between screening, diagnosis and intervention, and prevailing stigma and cultural misconceptions (*conceptions*) surrounding hearing impairment (Störbeck and Young 2016, Maluleke et al. 2023). Linguistic barriers, lack of available interpreters and lack of cultural competence compound these access issues in multilingual African contexts (Khoza-Shangase and Maluleke 2025). Studies from Nigeria and South Africa further demonstrate that stigma, skepticism about cochlear implants, and lack of structured post-implant rehabilitation exacerbate inequities in access (Jesuyajolu et al. 2023, Khoza-Shangase and Bent 2025).

#### Theme 6: system-level enablers: strategic opportunities for health system strengthening

The literature also identified several enablers and recommendations for improvement in the affordability and accessibility of EI services. Enablers include implementation of community-based care models to reach underserved populations (Olusanya 2006), family-centred models (Khoza-Shangase 2025), tech solutions like tele-audiology and mobile platforms (Bodington et al. 2021), and integration into maternal and child health systems (Kanji 2021). Recent evidence suggests that integration of newborn hearing screening into existing maternity services is a cost-efficient and feasible enabler in South Africa (Kanji and Kara 2013). Supportive policies and targeted funding schemes are also key.

#### Theme 7: structural cost drivers unique to Africa: addressing policy and procurement inequities

This review identified cost drivers that are uniquely shaped by African socioeconomic contexts. Cost drivers in Africa are shaped not only by economic scarcity but also by systemic and structural realities that differ markedly from high-income settings. A heavy reliance on donor funding creates vulnerability to shifting international priorities, leaving programmes unstable when donor cycles end (Fagan and Tarabichi 2018, Bodington et al. 2021). Urban–rural disparities further inflate costs: children in rural areas face higher travel and accommodation expenses for accessing audiology and EI services concentrated in major cities (Kerr et al. 2012). In many countries, weak integration between health and education sectors leads to duplication of costs and fragmented service delivery, limiting economies of scale (Störbeck and Young 2016). The absence of regional procurement systems for hearing aids and cochlear implants forces countries to import at small scale, increasing unit prices compared to pooled procurement models used elsewhere (WHO 2017). Moreover, political instability and competing health priorities [e.g. human immunodeficiency virus/acquired immunodeficiency syndrome (HIV/AIDS), tuberculosis (TB)], malaria) mean hearing health rarely receives dedicated financing, exacerbating inequities. Together, these uniquely African cost drivers highlight the need for sustainable, locally grounded financing mechanisms and structural reforms rather than reliance on externally driven models.

## Discussion

Interpreting these themes through a health systems lens reveals critical implications for Universal Health Coverage (UHC), financing, and service delivery reform—particularly as investments in EI align with long-term health and education sector cost-saving and equity objectives, as defined in UHC reforms and SDG 3.8. The findings above reveal layered and interacting barriers to effective and affordable EI in Africa. What follows is an interpretive commentary unpacking the broader implications. The findings highlight the significant role of cost considerations in shaping the landscape of EI for DHH children in Africa. The interplay between cost considerations and other systemic challenges creates a complex environment that often hinders access to timely and effective services. The consistent identification of high costs associated with various aspects of EI, from initial screening and diagnosis to the provision of assistive devices and ongoing therapy, highlights the substantial economic burden faced by families. This financial strain not only limits access but also exacerbates existing inequities, particularly affecting families in low-income and rural settings who rely on often under-resourced public healthcare systems. Newer qualitative and observational evidence from South Africa, Nigeria, and Kenya (e.g. Kanji and Kara 2013, Jesuyajolu et al. 2023, Ndegwa 2023) highlights how these financial challenges intersect with cultural, linguistic, and systemic barriers, compounding inequities in care. Cost considerations intersect with systemic inefficiencies, cultural beliefs, and geographic disparities to create a complex access environment. The repeated identification of cost as a limiting factor illustrates the structural neglect of EHC in policy and funding frameworks.

Despite the considerable financial obstacles, the evidence regarding the cost-effectiveness of EI and related technologies offers a compelling argument for increased investment. Publications from various African countries and global estimates suggest that interventions such as UNHS and the provision of hearing aids and cochlear implants can yield positive economic returns in the long term. By facilitating early identification and intervention, these programmes can potentially reduce the need for more expensive interventions and support services later in life, while also enhancing the individual's educational and vocational outcomes, thereby contributing to overall economic productivity. Compelling evidence of cost-effectiveness (e.g. Olusanya et al. 2009, WHO 2017) provides a strong case for scaling up EHC programmes. The economic return on EI investment extends beyond health outcomes, contributing to education, workforce inclusion, and poverty reduction. Evidence from Nigeria and Kenya further highlights how context-specific implementation models, such as community-based UNHS and education-focused interventions, can enhance cost-effectiveness when tailored to GDP thresholds (Olusanya 2006, Emmett et al. 2015, Ndegwa 2023).

The current funding landscape for EI in Africa is characterized by a mix of government funding, donor contributions, and significant out-of-pocket expenses for families. However, the sustainability and adequacy of these funding sources are often precarious. The lack of dedicated budgetary allocations for ear and hearing care in many African nations further compounds the financial challenges. This situation necessitates the exploration and implementation of more robust and sustainable funding models, including the integration of hearing health services into NHI schemes and the establishment of targeted subsidy programmes to alleviate the financial burden on families. South African studies (e.g. Kanji and Kara 2013, Khoza-Shangase and Bent 2025) reinforce how fragmented funding leaves families vulnerable, while also pointing to opportunities for leveraging NHI reforms to include hearing care.

The broader economic implications of unaddressed hearing loss extend beyond the individual and family levels, impacting national economies through increased healthcare and educational costs, reduced productivity, and societal costs associated with disability. Recognizing these substantial economic consequences provides a strong rationale for prioritizing investments in EI as a cost-saving measure in the long term. By addressing hearing loss early, societies can potentially mitigate these broader economic impacts and foster greater inclusion and participation of individuals with hearing loss. It is crucial to emphasize the importance of culturally sensitive and contextually appropriate EI models for Africa. The unique sociocultural norms, beliefs, and resource availability across the continent necessitate tailored approaches rather than the direct adoption of models developed in high-income countries. Engaging families and communities, respecting linguistic diversity, and integrating traditional support systems are essential for developing effective and sustainable interventions. The inclusion of studies explicitly addressing multilingualism and cultural competence (Jesuyajolu et al. 2023, Khoza-Shangase and Maluleke 2025) provides strong support for linguistically inclusive and Afrocentric EI approaches. Culturally responsive models—community-based, linguistically inclusive, and family-oriented—offer the best chance of scalable impact. These must be tailored to local health systems and reflect the Afrocentric contexts in which children and families live.

Building on the thematic insights presented, and to address the identified challenges and improve outcomes for DHH children in Africa, the following stakeholder-specific recommendations aim to translate evidence into policy and practice: (i) Policymakers should prioritize national investment in comprehensive EI programmes, implement mandatory UNHS, integrate hearing care into NHI and subsidy schemes, and establish targeted financial support for hearing aids, cochlear implants, and therapy; (ii) Healthcare providers must adopt community-based, linguistically inclusive, and culturally grounded service models, implement task-shifting strategies to expand the reach of services, and work collaboratively with families as active partners in EI planning and delivery; (iii) Researchers should expand health economic evaluations of EI models in diverse African settings, quantify the long-term developmental and economic returns of EI, and identify scalable and context-responsive financing solutions; and (iv) International organizations and donors should provide targeted financial and technical assistance, support infrastructure and workforce development, promote regional collaboration and knowledge exchange, and foster innovative public-private partnerships to strengthen EI systems across the continent. Addressing cost-related barriers requires not only financial investment but also strategic governance reforms, intersectoral coordination, and a commitment to decolonized, Afrocentric models of care that advance health equity and sustainability. By implementing the above strategies, stakeholders can collectively advance inclusive, integrated, and financially accessible EI systems for DHH children across Africa.

### Strengths and limitations

This review is subject to several limitations. The heterogeneity of study designs, geographic coverage, and definitions of cost made comparisons challenging. Many included studies lacked detailed economic modelling, limiting depth of cost-effectiveness interpretation. The underrepresentation of Central and North African countries constrains generalisability. Additionally, publication bias may favour studies with positive or measurable outcomes, and grey literature was excluded, possibly omitting relevant programme data. However, the inclusion of more recent qualitative and observational studies from South Africa, Nigeria, and Kenya (e.g. Kanji and Kara 2013, Jesuyajolu et al. 2023, Ndegwa 2023) strengthens the contextual validity of findings, particularly regarding cultural and linguistic dimensions of cost. Despite these limitations, the review offers a valuable synthesis of available evidence and identifies critical gaps for future research.

## Conclusion

This narrative review highlights the multidimensional, systemic, and policy-relevant influence of cost considerations on EI services for DHH children in Africa. While the high costs associated with screening, assistive devices, therapy, and follow-up care pose major barriers to access, findings indicate that EI services are not only economically justifiable but also cost-effective in the long term, particularly when implemented early and embedded within integrated health and education systems. The current funding environment—characterized by donor reliance, fragmented service delivery, and high out-of-pocket costs—highlights the urgent need to transition towards sustainable, publicly financed models that promote equity and universality. These must include increased government allocations, intersectoral financing mechanisms, and the inclusion of hearing services in essential health benefit packages under national insurance schemes.

In practical terms, this requires: (i) integrating UNHS into existing maternal and child health platforms such as immunization clinics to minimize additional infrastructure costs; (ii) establishing national subsidy schemes for hearing aids, cochlear implants, and therapy services to reduce out-of-pocket expenditure for families; (iii) adopting task-shifting approaches to train community health workers in basic hearing screening and counselling, thereby expanding service reach; (iv) creating pooled regional procurement mechanisms to lower device costs through bulk purchasing; and (v) mandating the inclusion of cost-effectiveness analyses in health policy planning to ensure evidence-based scale-up.

The expanded evidence base from South Africa, Nigeria, and Kenya also reinforces the importance of designing linguistically inclusive, culturally responsive, and community-based EI systems that reflect African realities. By implementing these concrete strategies, African health systems can move towards inclusive, effective, and affordable EI programmes that improve developmental outcomes for DHH children while also generating long-term economic and social benefits.

- This paper looks at the costs families face when trying to get early help for DHH children in Africa.
- We reviewed 26 research studies to understand the financial and system challenges that stop many children from getting the support they need early in life.
- Families often must pay large amounts themselves for hearing tests, hearing aids, or therapy, which many cannot afford.
- The paper shows that early support can save money in the long run by helping children do better in school and in life.
- We suggest practical steps such as including hearing tests in baby check-ups, reducing device costs through group purchasing, offering government subsidies for hearing aids, and training community health workers to provide local support.

## Supplementary Material

czaf074_Supplementary_Data

## Data Availability

Data supporting the findings of this study are available within the paper.

## Reflexivity statement

The author is an audiologist and public health researcher and audiology based in Africa, with professional and research expertise in early hearing detection and intervention (EHDI). I acknowledge that this positionality shaped my focus on equity, access, and sustainability in African health systems. To minimize bias, I documented search strategies, applied structured thematic synthesis, and engaged in reflexive discussions to ensure balanced and contextually relevant interpretation.
