# Supplementary material for: A narrative review on cost considerations in early intervention for deaf and/or hard-of-hearing children in Africa
Source: Health Policy Plan. 2025 Oct 8;41(2):286–98. doi: 10.1093/heapol/czaf074 (PMC13006968; doi:10.1093/heapol/czaf074)
Supplement: czaf074_Supplementary_Data [file czaf074_supplementary_data.docx]

**Appendices**

**Appendix 1:** Search Strategy (Example: PubMed)

| **Concept** | **Keywords / Search Terms** |
| --- | --- |
| Population | “deaf” OR “hard of hearing” OR “hearing loss” OR “hearing impairment” OR “hearing disabled” |
| Intervention | “early intervention” OR “early hearing detection” OR “EHDI” OR “screening” OR “diagnosis” OR “rehabilitation” |
| Cost / Economics | “cost” OR “cost analysis” OR “economic evaluation” OR “cost-effectiveness” OR “affordability” OR “resource allocation” OR “financing” |
| Geography | “Africa” OR names of individual African countries (e.g., “South Africa” OR “Nigeria” OR “Kenya” OR “Ghana”) |

**PubMed example string:**

(“deaf” OR “hard of hearing” OR “hearing loss” OR “hearing impairment”)

AND (“early intervention” OR “early hearing detection” OR “EHDI”)

AND (“cost” OR “economic evaluation” OR “financing” OR “resource allocation”)

AND (“Africa” OR “South Africa” OR “Nigeria” OR “Kenya” OR “Ghana”)

**Databases searched**: PubMed, Scopus, Web of Science, Google Scholar.

**Limits applied**: Peer-reviewed journal articles published between 2000–2024, English language.

**Appendix 2:** Appraisal of Included Studies

| **#** |  | **Citation** | **Methodological Clarity** | **Relevance to Objectives** | **Contextual Fit (Africa)** | **Notes** |
| --- | --- | --- | --- | --- | --- | --- |
| 1 |  | Störbeck and Young (2016) | Moderate | High | High | Strong on systemic/cultural barriers; limited direct costing. |
| 2 |  | Emmett and Francis (2015) | High | High | High | Rigorous DALY-based analysis; strong SSA applicability. |
| 3 |  | Fagan and Tarabichi (2018) | Moderate | Moderate | Medium | Ethical/policy insights; less empirical evidence. |
| 4 |  | Bodington et al. (2021) | Moderate | High | High | Useful synthesis on cochlear implants feasibility in LMICs. |
| 5 |  | Fagan (2012) | Moderate | Moderate | High | Emphasises training gaps; frames global responsibility. |
| 6 |  | Kerr et al. (2012) | High | High | High | Detailed cost estimates for cochlear implants in South Africa; strong contextual insight. |
| 7 |  | Olusanya et al. (2009) | High | High | High | DALY-based UNHS cost-effectiveness in LMICs. |
| 8 |  | Smith (2013) | High | High | High | Comprehensive doctoral analysis of SA costs and policy gaps. |
| 9 |  | WHO & World Bank (2011) | Moderate | High | High | Policy-level evidence; broad but Africa included. |
| 10 |  | Emmett et al. (2015) | High | High | High | Strongly promotes cochlear implants and educational investment for children in SSA |
| 11 |  | Baltussen & Smith (2009) | High | High | High | WHO-CHOICE modelling applicable to Africa. |
| 12 |  | Maluleke (2022) | Moderate | High | High | Strong advocacy for EHDI cost evaluation in South Africa. |
| 13 |  | Ndegwa et al. (2024) | Moderate | High | High | Kenya policy brief with ROI evidence. |
| 14 |  | Khoza-Shangase (2025) | High | High | High | Afrocentric/decolonial lens; strong alignment with objectives. |
| 15 |  | WHO (2017) | High | High | High | Global costing with African implications. |
| 16 |  | Swanepoel et al. (2009) | Moderate | High | High | South Africa-specific service/system barriers; limited costing detail. |
| 17 |  | Casoojee et al. (2024) | Moderate | High | High | Private vs. public model cost trade-offs in South Africa. |
| 18 |  | Swanwick et al. (2022) | High | High | High | Rich qualitative caregiver evidence in Ghana; highlights ECCE barriers. |
| 19 |  | Olusanya et al. (2008) | High | High | High | Cost per case analysis; highly relevant for LMICs. |
| 20 |  | Sharma et al. (2019) | High | Moderate | Medium | Global review; limited African focus. |
| 21 |  | Kanji & Kara (2013) | High | High | High | Empirical South African cost data on newborn hearing screening; feasible and low-cost. |
| 22 |  | Petrocchi-Bartal et al. (2025) | Moderate | High | High | Identifies Early Intervention policy–practice gaps; limited costing. |
| 23 |  | Olusanya (2006) | Moderate | High | High | Foundational argument for EHDI in LMICs; feasibility lens. |
| 24 |  | Ndegwa (2023) | Moderate | High | High | Kenya cochlear implants delays study; strong evidence on systemic/financial barriers. |
| 25 |  | Jesuyajolu et al. (2023) | Moderate | High | High | Nigerian cochlear implants scoping review; highlights cost, rehab, and workforce gaps. |
| 26 |  | Khoza-Shangase & Bent (2025) | High | High | High | Parental decision-making on cochlear implants in South Africa; highlights financial and cultural barriers. |

Key: DALY: Disability-Adjusted Life Year; SSA: Sub-Saharan Africa; LMICs: Low- and Middle-Income Countries; UNHS: Universal Newborn Hearing Screening; EHDI: Early Hearing Detection and Intervention; ROI: Return on Investment; ECCE: Early Childhood Care and Education
